# Altered Gut Microbiota Diversity and Composition in Chronic Urticaria

**DOI:** 10.1155/2019/6417471

**Published:** 2019-11-11

**Authors:** Tao Lu, Yanxia Chen, Yangmin Guo, Jiayu Sun, Weitao Shen, Mengsi Yuan, Shuping Zhang, Ping He, Xiaoyang Jiao

**Affiliations:** ^1^The First Affiliated Hospital of Shantou University Medical College, China; ^2^Biology and Genetic Department of Shantou University Medical College, China; ^3^The Second Affiliated Hospital of Shantou University Medical College, China

## Abstract

**Background:**

The pathogenesis of chronic urticaria (CU) is closely related to imbalances in immunity. The gastrointestinal microflora provides a vast and continuous stimulation for the immune system. However, the composition and diversity of gut microflora in CU patients are rarely reported.

**Methods:**

10 CU patients and 10 healthy individuals were selected in this study, and their intestinal microbiome was detected by 16S rRNA sequencing. The data were analyzed using R language software.

**Results:**

392 bacterial OTUs were common in the CU and healthy groups, but there were 159 OTUs particularly existing in the CU group, while 87 OTUs only were observed in healthy individuals. The bacterial diversity was reduced in CU patients compared with healthy individuals. The principal component analysis (PCA) and principal coordinate analysis (PCoA) revealed that the bacterial cluster in CU patients and the healthy controls were divided into different branches. Pathogenic strains including *Escherichia coli* were significantly higher in CU, while *Faecalibacterium prausnitzii*, *Prevotella copri*, and *Bacteroides* sp. were significantly lower in CU when compared with the healthy controls. CU patients with a high abundance of *Escherichia coli* had no ideal effect for probiotic therapy.

**Conclusion:**

Our results demonstrated that the microbial composition was significantly different between CU patients and the healthy individual, which may be the reason leading to the various outcomes of probiotic treatment.

## 1. Introduction

Urticaria is one of the most common diseases observed in a dermatologic practice, characterized by the development of wheals, angioedema, or both. Acute urticaria (AU) is mostly related to an allergic or pseudoallergic reaction to food, drugs, or infections. Chronic urticaria (CU) is a spontaneous or inducible disorder defined as persistent urticaria longer than 6 weeks in duration and without an identifiable cause [1]. Some patients with AU may turn into CU though the treatment protocol is the same. Compared to AU, CU is a more complex disease that may relate to imbalances in immunity, inflammation, and coagulation [2]. Although extensive studies have been done, the pathogenesis of CU is still largely unknown.

The skin is the largest organ of the human body that harbors several hundreds of resident microorganisms. These microbes are collectively referred to as the skin microbiota that is fundamental to skin physiology and immunity [3]. Studies have shown that shifts in the skin microbiota are associated with skin diseases [4, 5]. Atopic dermatitis flares are associated not only with blooms of *Staphylococcus aureus* but also with significant decreases in microbial diversity [6]. Besides skin microbiome, changes in the human gut microbiome have been reported in atopic dermatitis and allergy. The latest study revealed that disturbances in gut microbiota composition and/or activity (dysbiosis) might contribute to the pathogenesis of allergic diseases [7]. From infancy, the gastrointestinal tract has been provided a vast and continuous source for bacterial stimulation of the immune system [8]. Gut-colonizing bacteria, reacting with Toll-like receptors located on the intestinal epithelium and dendritic cells, stimulate the signaling pathways of immune effector cells, including macrophages, B cells, NK cells, and T cells (helper/cytotoxic/regulatory T cells) [9]. Therefore, gut microbiota plays an important role in the evolution and regulation of the immune system [10]. Reduced exposure to microbes in early childhood may affect the natural development of the immune system or immune tolerance, resulting in increased susceptibility to allergic diseases [11]. An imbalance in the intestinal microbiome is influencing the development of allergic disease [8]. With the gut microbiota reduced exposure to the immunologic system, a notable increase in the incidence and prevalence of allergic as well as autoimmune and inflammatory disorders has been reported worldwide [12]. Several studies demonstrate the role of gut microbiota in allergic diseases such as asthma [13], food allergy [14], and atopic eczema [15]. Most activated or memory T cells reside in tissues that are constitutively colonized by commensals such as the skin and the GI tract. At steady state, most IL-17 (Th17) and IFN*γ* (Th1) T cells are found in the GI tract and develop from signals derived from the microbiota [10, 16, 17]. Aberrant expression cytokines are frequently observed in patients with CU [18]. In the previous study, Th1 and Th2 cytokine expressions were closely correlated with urticarial disease severity, indicating that Th1 and TH2 imbalance participates in the pathogenesis of urticaria [19]. Treg cells regulate the immune response and are characterized by a specific cytokine profile. However, the mechanism that induced the aberrant cytokine expression in CU is still unclear.

Nowadays, regulation of the immune system through gut microbiota is supposed to affect the CU. Whether intestinal flora imbalance results in the susceptibility to CU, few reports demonstrated it. Recently, the changes in bacterium *Akkermansia muciniphila*, *Faecalibacterium prausnitzii*, *Clostridium leptum*, and *Enterobacteriaceae* were reported in CU patients [2]. Unfortunately, the whole intestinal microbiome in CU patients is rarely being clarified. Most metagenomic cataloging of the human microbiome has focused on species composition. Recent work demonstrates that, even within the same species, different strains can differ markedly in their effects on the host [20]. Strain-level differences have been largely unexplored and remain a frontier for studies of the microbiota [3]. Intestinal microbiome in the different populations may be diverse and individually specific, which related to race genetics, lifestyle, diet habit, antibiotics, etc. Our study tries to provide a better understanding of whether differences in fecal microbiota correlate with the occurrence of CU, which facilitates future efforts in understanding the possible pathogenesis behind CU-related bacterial targets and probiotic treatment.

## 2. Methods

### 2.1. Study Design and Participants

The ethics committee of the Shantou University Medical College approved this study, and written informed consent was obtained from all the subjects before participation. All the study procedures and the participants were following the Declaration of Helsinki (1964) and its later amendments. The evaluation of the patients was based on history and physical examination. The urticaria activity score (UAS) was calculated under EAACI/GA2LEN/EDF guidelines [1]. Urticaria was diagnosed and assessed according to the American Academy of Allergy, Asthma, and Immunology criteria. Accordingly, 10 patients with acute urticaria as the first symptom but turned to CU were enrolled in the study. 10 healthy individuals were enrolled as controls. Fecal samples of approximately 5 g were obtained from each subject and stored at -80°C until DNA extraction.

### 2.2. DNA Extraction from Feces and 16S rRNA Amplicon Sequencing

Approximately 5 g of fresh stool samples was obtained from January 1 to December 31, 2017. The subjects were allowed to excrete the feces into a clean container and to avoid contaminating the urine and the toilet sidewalls, and the samples were sent to the laboratory immediately. A sterile spoon was used to remove the stool sample from the feces and distribute it to three sterile tubes (5 g/tube) and stored at -80 degrees Celsius. The sample should be protected from repeated freezing and thawing. DNA was extracted from 200 ± 20 mg of feces using a QIAamp DNA stool mini kit (Qiagen, Hilden, Germany) according to the manufacturer's instructions. The purity of extracted genomic DNA was confirmed via spectrophotometric analyses (A260/280 ratio of 1.8). Then, all the qualified DNA was used to construct a library (or libraries). For gDNA, we use fusion primer with a dual index and adapters for PCR. In both cases, only the qualified library can be used for sequencing. Build a library with qualified samples: Paired-end sequencing reads were obtained as demultiplexed libraries per sample. Briefly, 16S amplicon PCR forward primer (V3 region 341F): ACTCCTACGGGAGGCAGCAG, 16S amplicon PCR reverse primer (V4 region 806R): GGACTACHVGGGTWTCTAAT, (V4 region 515F): GTGCCAGCMGCCGCGGTA A, and (V4 region 806R): GGACTACHVGGGTWTCTAAT were used.

### 2.3. Bioinformatic Analysis Workflow

Paired-end reads with overlap were merged to tags. And tags were clustered to OTU at 97% sequence similarity. Taxonomic ranks were assigned to OTU representative sequence using the Ribosomal Database Project (RDP) Native Bayesian Classifier v.2.2. The indices are calculated by Mothur (v1.31.2). Alpha diversity, beta diversity, and the different species screening were analyzed based on OTU and taxonomic ranks. After filtering raw data, we obtained a dataset consisting of a total of 833286 high-quality 16S rRNA gene sequences, with an average of 41664 ± 382 (S.E.) sequences per sample. Within the dataset, we identified a total of 638 OTUs, based on 97% sequence similarity (equal to bacterial species level). Bioinformatic analyses were performed to the obtained data.

### 2.4. Statistical Analyses

Categorical and continuous variables were tested by the chi-squared test and two-sample *t*-test, respectively. In multivariate analysis, significant variables from univariate analysis were selected and manually entered to the model step by step. Data were analyzed using SPSS 17.0 software, and all *P* values represent two-sided statistical tests. Bioinformatic analyses were done by R language software.

## 3. Results

### 3.1. Clinical Features of the CU and the Controls

The demographic and laboratory indices of CU and control groups were presented in Table 1. 10 patients with CU (3 men and 7 women; median age: 33 years old; range: 7-62 years old) were included in the study. For comparison, the control group consisted of 10 healthy subjects (5 men and 5 women; median age: 43 years old; range: 34.5-51.25 years old) who had not taken any medicine for at least 2 weeks preceding the study. There is no significant difference in age, as well as other lab parameters except the WBC and CRP. WBC and CRP in the CU group were significantly higher than that in the control, indicating the infectious status of CU patients (*P* < 0.05).

### 3.2. Microflora Diversity in CU Patients and Healthy Individuals

**Figure 1:**
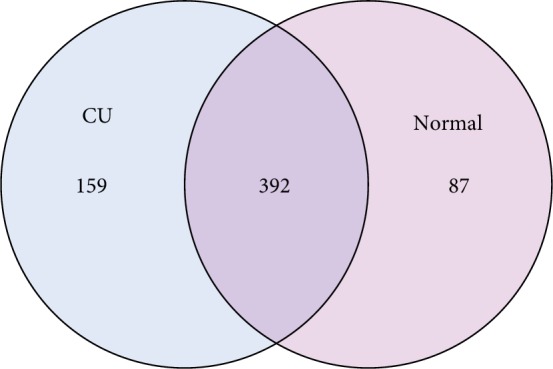
Bacteria OTUs in CU and healthy groups.

**Figure 2:**
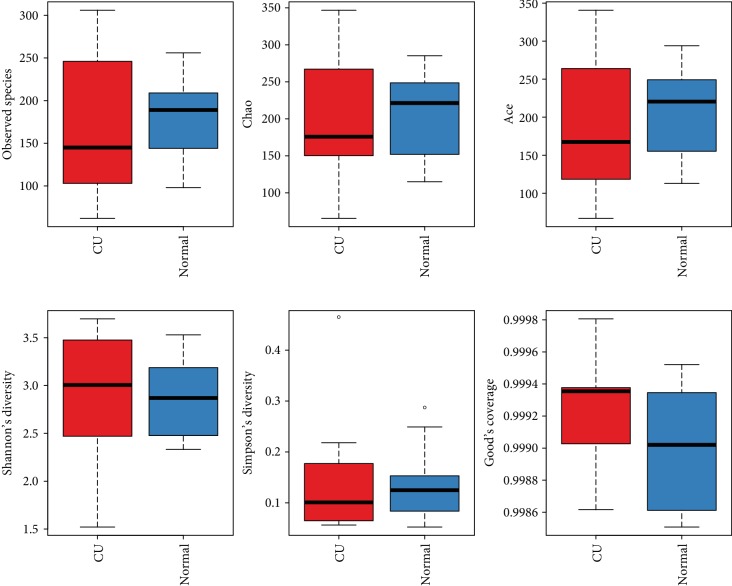
Boxplot was used to visually display the differences of the alpha diversity (Observed species, Chao, Ace, Shannon, and Simpson indices) between CU and healthy groups.

For clarifying the features of OTUs between various groups, the Venn diagram analysis (OTU Venn graph results) was performed using R language software. 392 OTUs of bacteria were common in the CU and healthy groups. There were 87 OTUs only observed in healthy fecal samples, while 159 OTUs are particularly existing in the CU group (Figure 1). The microflora diversity was compared in CU patients and healthy individuals, including *α* and *β* diversity that estimate gut microbiota richness and evenness [21]. *α* diversity was based on the indices Observed species, Chao, Ace, Shannon, and Simpson indices. In this study, Sobs, Chao, Ace, and Simpson were lower in CU than in normal, indicating that community richness of gut flora in CU is lower than that in normal. The Shannon index is used to estimate microbial diversity in samples. The greater the Shannon value, the higher the community diversity. A higher Shannon value was found in the CU group, which is inconsistent with other indices, and the possible reason may be the sample bias. The species diversity index in the CU group was lower than those in the normal controls, but the difference did not reach the statistical significance; the reason may be due to the small simple size. Boxplot was used to visually display the differences of the *α* diversity among groups (Figure 2).

**Figure 3:**
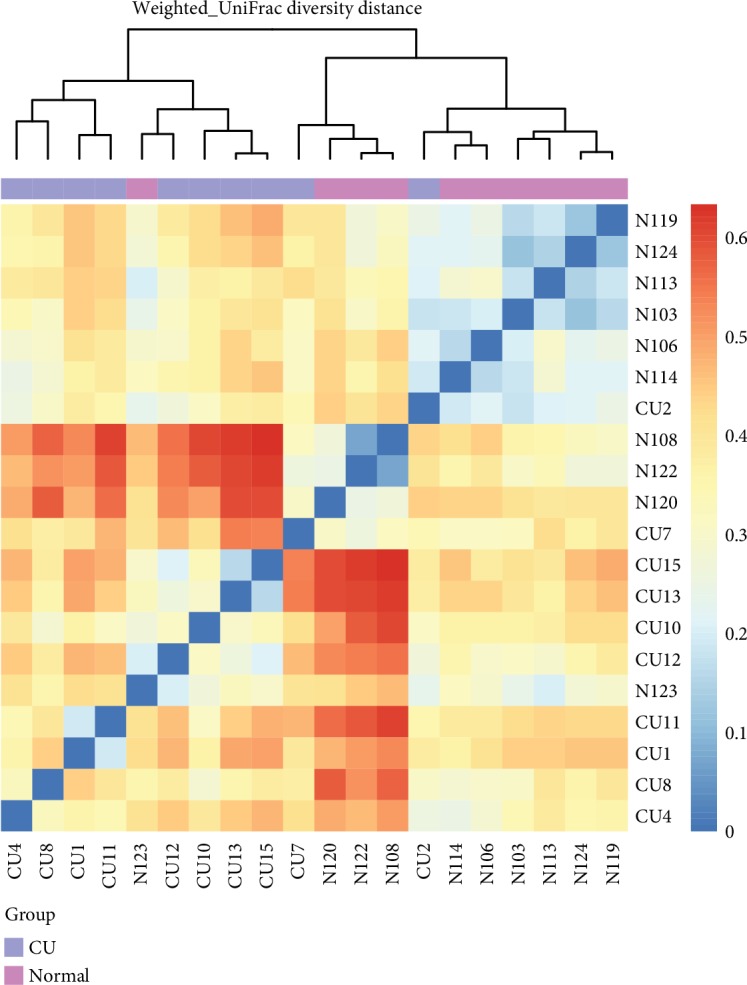
Heatmap revealed *β* diversity in CU and healthy individuals. The samples with similar *β* diversity were clustered together.

**Figure 4:**
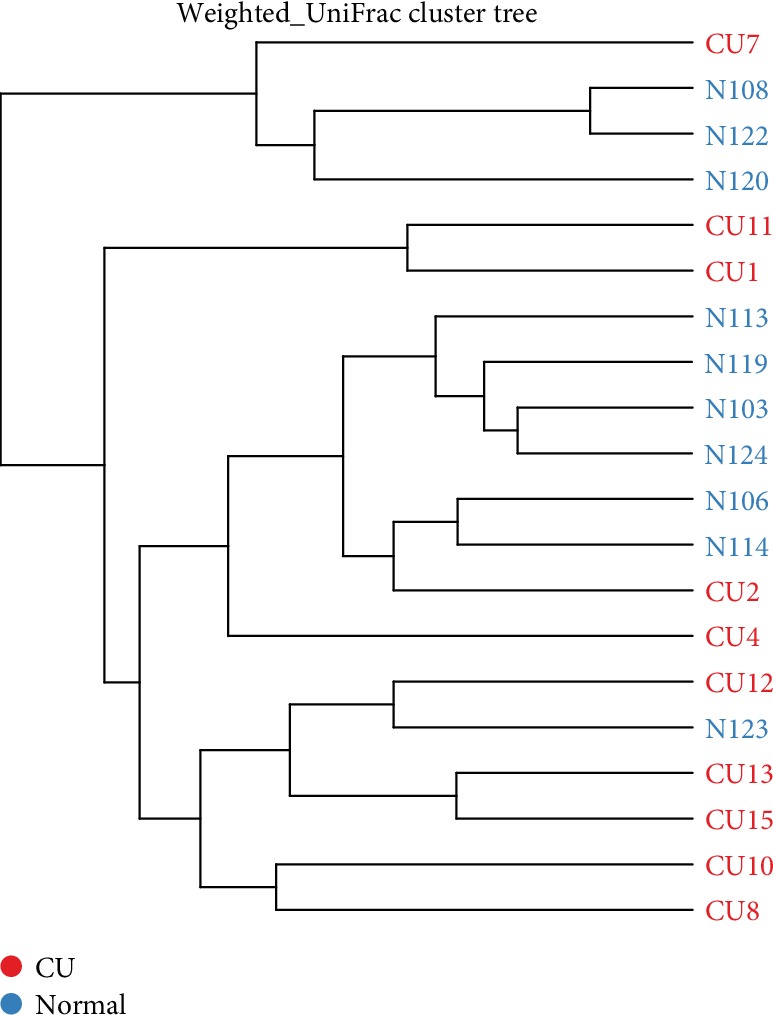
UPGMA clustering tree showed that different branches accumulated in CU patients and healthy individuals, respectively.

**Figure 5:**
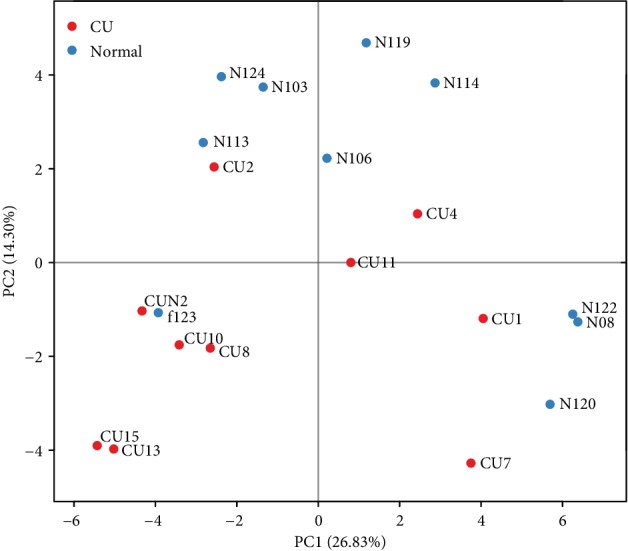
The PCA results indicate that bacteria in the healthy control group were clustered apart from the CU group. This suggested that abundances of classified OTUs may differentiate CU from the healthy matched controls.

**Figure 6:**
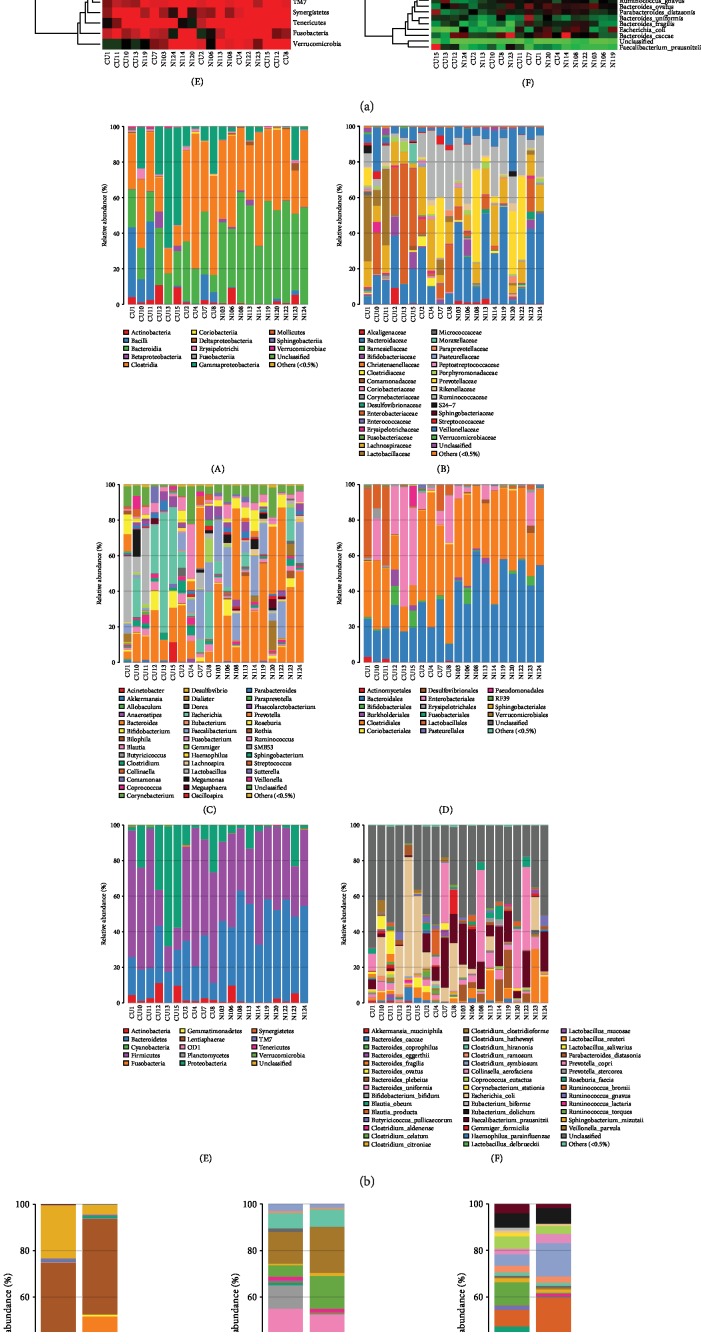
Heatmap revealed the intestinal flora of CU patients and healthy individuals in the class, family, genus, order, phylum, and species (according to a–c).

**Figure 7:**
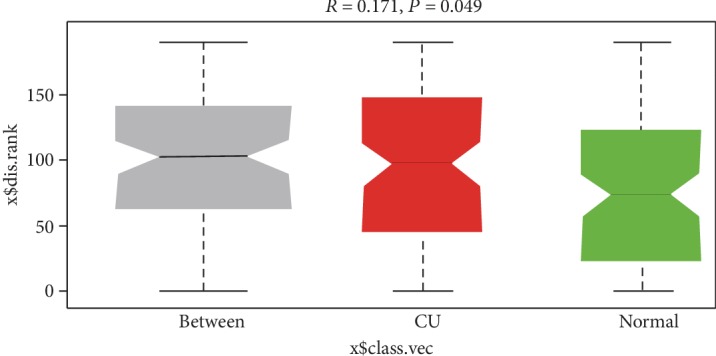
ANOSIM analysis was done for the comparison of *β* diversity; our results indicated that the difference between the groups is greater than the difference within the group; that is, the difference between the groups is significant.

The differences in microbial composition (*β* diversity) were assessed, which was used to evaluate differences of samples in species complexity. By using unweighted UniFrac *β* diversity analyses, the results revealed that *β* diversity in the control group was significantly different from the CU group (*P* < 0.01). Beta matrix heatmap was used to reflect the similarity of samples. Samples with similar *β* diversity are clustered together (Figure 3). In order to further analyze the differences in bacterial community complexity between samples, UniFrac software was used to analyze the differences of samples, which include UPGMA clustering tree analysis (Figure 4), principal component analysis (PCA), and principal coordinate analysis (PCoA) (Figures 5 and 6). The results showed that CU patients and normal controls were divided into different branches in the cluster map (without considering OTU abundance), demonstrating that the difference of flora was obvious between CU patients and normal controls. ANOSIM analysis was done for the comparison of *β* diversity; our results indicated that the difference between the groups is greater than the difference within the group; that is, the difference between the groups is significant (Figure 7).

### 3.3. Comparison of Gut Microflora between CU Patients and Healthy Controls at Different Levels

The taxonomic composition distribution histograms in two groups were shown at the levels of phylum, order, class, family, genus, and species, respectively. The bacterial OTUs in different samples were also summarized in a profiling table or histogram. The top discriminatory OTUs that are distinctive between CU and non-CU were compared based on taxonomy-independent hierarchical classification. At the levels of phylum, the dominant flora was *Bacteroidetes*, *Firmicutes*, *Actinobacteria*, and *Proteobacteria*. *Bacteroidetes* was higher in controls than in CU; contrarily, *Actinobacteria* and *Proteobacteria* were higher in CU than in controls. In the order level, *Enterobacteriales*, *Lactobacillales*, and *Pseudomonadales* in the CU group were significantly higher than those in controls. In the genus level, *Veillonella*, *Sutterella*, *Streptococcus*, *Clostridium*, and *Escherichia* in the CU group were significantly higher than those in the healthy group.

On the contrary, *Faecalibacterium*, *Prevotella*, and *Lachnobacterium* in CU were significantly lower than those in the normal. In the species level, *Escherichia coli* was significantly higher in CU than in the normal, while *Faecalibacterium prausnitzii*, *Prevotella copri*, *Bacteroides fragilis*, and *Bacteroides plebeius* were significantly lower in CU than in the normal. Our results demonstrated that the microbial composition was significantly different between CU patients and the healthy individual at the genus level.

## 4. Discussion

Imbalanced microbiota diversity should be considered as one of the most important underlying causes of allergic disease [22]. The study indicated that decreased bacterial diversity increased the risk of allergic sensitization, allergic rhinitis, and peripheral blood eosinophilia; moreover, reduced diversity in early age will cause increased susceptibility of allegoric disease, i.e., asthma in later stages [8]. In this study, decreased diversity was observed in CU patients. Although the mechanism of decreased microbiota diversity associated with the etiology of CU is not clear, alterations in gut bacterial diversity could disrupt mucosal immunological tolerance by promoting Treg cells reacting to dietary antigens [23].

Gut microbiota disturbances contribute to the pathogenesis of allergic diseases [7]. *Firmicutes* and *Bacteroidetes* are the dominant phyla in hundreds of bacterial and archaeal species of the gut harbors [24, 25], followed by *Actinobacteria* and *Verrucomicrobia* [9]. In our study, the dominant phyla was *Bacteroidetes*, which was higher in normal controls. Specifically, *Bacteroides fragilis*, *Bacteroides plebeius*, and *Bacteroides uniformis* were significantly higher in the normal group than in CU, indicating that the reduction of *Bacteroides* might be related to the pathogenesis of CU; presently, the role of Bacteroides sp. is needed to be further confirmed in allergic disorders. Increased *Proteobacteria* in the gut drastically enhanced the permeability of the normally sterile mucus inner layer to the more penetrable region, resulting in bacterial infiltration into the intestinal inner layer close to the epithelium [24]. The inflamed epithelium with impaired barrier function has been associated with atopic eczema, celiac disease, and Crohn disease [26, 27].

An increase in the abundance of *Actinobacteria* and *Proteobacteria* was found in our patients with CU; the similar mechanism may exist in CU patients with a high abundance of *Proteobacteria*. *Enterobacteriaceae* is the main branch of *Proteobacteria* containing genera of *Escherichia coli* (*E. coli*), *Klebsiella* spp., and *Proteus* spp. Among them, *E. coli* and *Klebsiella spp.* have the potential for overgrowth and intestinal domination during dysbiosis [28]. In our study, a higher abundance of *E. coli* observed in CU patients may have important clinical significance in CU etiology. *Faecalibacterium prausnitzii* belongs to the *Clostridium leptum* group from the *Firmicutes* phylum. Lower amounts of *Faecalibacterium* species are observed in allergy and atopic dermatitis [29]. In our study, *Faecalibacterium prausnitzii* was also significantly lower in CU than in the normal. Besides these bacterial strains, the distinct microbiome profiles between CU patients and healthy individuals were observed. Our observations of differentially abundant species and strains between the CU patients and healthy individuals revealed that some bacterial patterns determine the etiology of CU. The enrichment of *Enterobacteriaceae* and decreases in *Lactobacillus* and *Bifidobacterium* species were observed in patients with AD [30, 31]. Then, gut microbiota may be a target for improving outcomes in subjects affected or at risk for allergic diseases. The protective role of these bacteria against atopic diseases and inflammatory states has been demonstrated [32]. Specific bacterial genera including *Lactobacillus* and *Bacteroides* as well as their microbial metabolites, i.e., short-chain fatty acids, confer protection against allergy and asthma [22, 33].

CU has been estimated to be as high as 0.5% to 1% of the population, and up to 20% of persons with CU may continue to have symptoms 20 years after the onset [34]. Many patients fail to achieve satisfactory control with an antihistamine alone, but no alternative therapy has been fully accepted or received regulatory approval [1]. In the present clinical practice, modification of gut microbiota via the provision of probiotics and/or prebiotics is the most extensively studied strategy [35]. However, the microbiota composition and diversity reduction on the pathogenesis of CU are unclear; the related study may provide a guide for the probiotic's treatment. In this study, CU patients were treated with probiotics; however, the therapeutic efficacy is different. Distinct microbiome profiles in patients may be the main reason for inconsistent therapeutic efficacy. Of note, the therapeutic efficacy of patients with a higher abundance of *E. coli* was poor (data are not shown). Further study with large simple size is needed to reveal the direct relation between the abundance of *E.coli* and the efficacy of the probiotic.

Nowadays, *Lactobacillus* in the prevention and treatment of the allergic and inflammatory diseases was reported [36, 37]. However, the results of clinical trials are inconsistent. There was no effect of *Lactobacillus* administration on reducing the risk of wheezing/asthma [35]. The risk is that pooling data from different genera, species, strains, and doses of probiotics obtained in different settings and/or populations, presumably with variations in their native intestinal microbiota, may result in misleading conclusions. Therefore, some studies indicate that current guidelines on the use of probiotics for preventing eczema in infants at high risk should be revised and be more specific concerning which strain(s) to use [35]. A debate is raising about “Which probiotic(s) should be used to reduce the risk of eczema? What is the dose of an effective probiotic [38]?” and did or did not recommend the use of probiotics for reducing the risk of allergy in children [39, 40].

Accurate analysis of the intestinal flora will facilitate the establishment of evaluating the system for assessing allergic patients who are appropriate for the probiotic treatment. Our results indicated that CU patients with particular bacterial flora might have different therapeutic efficacies after probiotic treatment. Therefore, understanding the composition and overall structure of gut microbiota in CU patients before therapeutic protocol enactment is crucial for the therapeutic efficacy. The gut microbial composition may be a cost-efficient and noninvasive biomarker for treatment proevaluation. Large sample size studies are needed to provide a species of bacterial patterns of CU patients.

## Figures and Tables

**Table 1:** Clinical characters of CU patients and healthy controls.

Variable	Experimental (*n* = 10)	Control (*n* = 10)
Age	33 (7-62)	43 (34.5-51.25)
Gender (male : female)	3 : 7	5 : 5
WBC (10*E* + 9/*L*)	10.86 (4.98-16.73)	6.90 (6.15-7.63)
Eo (%)	0.04 (0.001-0.08)	0.16 (0.09-0.28)
RBC	4.53 (3.62-5.43)	4.74 (4.52-5.26)
Hb (g/L)	111.5 (80-143)	142.50 (133-152.50)
PLT	231.5 (155-308)	239.50 (217.75-261.50)
CRP	59.55 (0.1-119)	4.00 (0.01-8.00)
Glu	6.34 (3.67-9.01)	5.15 (5.03-5.36)
Ca^2+^	2.16 (1.97-2.34)	
IgG	10.95 (10.40-11.50)	11.56 (7.51-15.60)
IgM	2.08 (1.35-2.81)	1.75 (0.46-3.04)
IgA	0.97 (0.62-1.32)	2.68 (0.82-4.53)
C3	0.86 (0.81-0.90)	1.16 (0.79-1.52)
C4	0.19 (0.13-0.24)	0.27 (0.16-0.38)

^∗^WBC: white blood cell; Eo: percentage of eosinophils; RBC: red blood cell; Hb: hemoglobin; PLT: platelet; CRP: C-reactive protein; Glu; glucose; Ca^2+^: calcium; IgG: immunoglobulin G; IgM: immunoglobulin M; IgA: immunoglobulin A; C3: complement 3; C4: complement 4.

## Data Availability

The data used to support the findings of this study are available from the corresponding author upon request.

## References

[1] Zuberbier T., Asero R., Bindslev-Jensen C. (2009). EAACI/GA2LEN/EDF/WAO guideline: definition, classification and diagnosis of urticaria. *Allergy*.

[2] Nabizadeh E., Jazani N. H., Bagheri M., Shahabi S. (2017). Association of altered gut microbiota composition with chronic urticaria. *Annals of Allergy, Asthma & Immunology*.

[3] Chen Y. E., Fischbach M. A., Belkaid Y. (2018). Skin microbiota–host interactions. *Nature*.

[4] Alekseyenko A. V., Perez-Perez G. I., De Souza A. (2013). Community differentiation of the cutaneous microbiota in psoriasis. *Microbiome*.

[5] Fitz-Gibbon S., Tomida S., Chiu B. H. (2013). Propionibacterium acnes strain populations in the human skin microbiome associated with acne. *The Journal of Investigative Dermatology*.

[6] Kong H. H., Oh J., Deming C. (2012). Temporal shifts in the skin microbiome associated with disease flares and treatment in children with atopic dermatitis. *Genome Research*.

[7] Pascal M., Perez-Gordo M., Caballero T. (2018). Microbiome and allergic diseases. *Frontiers in Immunology*.

[8] Bisgaard H., Li N., Bonnelykke K. (2011). Reduced diversity of the intestinal microbiota during infancy is associated with increased risk of allergic disease at school age. *Journal of Allergy and Clinical Immunology*.

[9] Tlaskalová-Hogenová H., Štěpánková R., Hudcovic T. (2004). Commensal bacteria (normal microflora), mucosal immunity and chronic inflammatory and autoimmune diseases. *Immunology Letters*.

[10] Belkaid Y., Hand T. W. (2014). Role of the microbiota in immunity and inflammation. *Cell*.

[11] Rook G. A., Lowry C. A., Raison C. L. (2013). Microbial ‘Old Friends’, immunoregulation and stress resilience. *Evolution, Medicine, and Public Health*.

[12] Rook G. A., Raison C. L., Lowry C. A. (2014). Microbiota, immunoregulatory old friends and psychiatric disorders. *Advances in Experimental Medicine and Biology*.

[13] Arnold I. C., Dehzad N., Reuter S. (2011). Helicobacter pylori infection prevents allergic asthma in mouse models through the induction of regulatory T cells. *The Journal of Clinical Investigation*.

[14] Ling Z., Li Z., Liu X. (2014). Altered fecal microbiota composition associated with food allergy in infants. *Applied and Environmental Microbiology*.

[15] Hulshof L., Land B., Sprikkelman A., Garssen J. (2017). Role of microbial modulation in management of atopic dermatitis in children. *Nutrients*.

[16] Gaboriau-Routhiau V., Rakotobe S., Lécuyer E. (2009). The key role of segmented filamentous bacteria in the coordinated maturation of gut helper T cell responses. *Immunity*.

[17] Ivanov I. I., Frutos Rde L., Manel N. (2008). Specific microbiota direct the differentiation of IL-17-producing T-helper cells in the mucosa of the small intestine. *Cell Host & Microbe*.

[18] Puxeddu I., Italiani P., Giungato P. (2013). Free IL-18 and IL-33 cytokines in chronic spontaneous urticaria. *Cytokine*.

[19] Lu T., Jiao X., Si M. (2016). The correlation of serums CCL11, CCL17, CCL26, and CCL27 and disease severity in patients with urticaria. *Disease Markers*.

[20] Byrd A. L., Deming C., Cassidy S. K. B. (2017). Staphylococcus aureusandStaphylococcus epidermidisstrain diversity underlying pediatric atopic dermatitis. *Science Translational Medicine*.

[21] Youssef N. H., Elshahed M. S. (2008). Species richness in soil bacterial communities: a proposed approach to overcome sample size bias. *Journal of Microbiological Methods*.

[22] Panzer A. R., Lynch S. V. (2015). Influence and effect of the human microbiome in allergy and asthma. *Current Opinion in Rheumatology*.

[23] Kim K. S., Hong S. W., Han D. (2016). Dietary antigens limit mucosal immunity by inducing regulatory T cells in the small intestine. *Science*.

[24] The Human Microbiome Project Consortium (2012). Structure, function and diversity of the healthy human microbiome. *Nature*.

[25] Faith J. J., Guruge J. L., Charbonneau M. (2013). The long-term stability of the human gut microbiota. *Science*.

[26] Arrieta M. C., Bistritz L., Meddings J. B. (2006). Alterations in intestinal permeability. *Gut*.

[27] Pike M. G., Heddle R. J., Boulton P., Turner M. W., Atherton D. J. (1986). Increased intestinal permeability in atopic eczema. *The Journal of Investigative Dermatology*.

[28] Taur Y., Pamer E. G. (2013). The intestinal microbiota and susceptibility to infection in immunocompromised patients. *Current Opinion in Infectious Diseases*.

[29] Koga Y., Tokunaga S., Nagano J. (2016). Age-associated effect of kestose on Faecalibacterium prausnitzii and symptoms in the atopic dermatitis infants. *Pediatric Research*.

[30] Candela M., Rampelli S., Turroni S. (2012). Unbalance of intestinal microbiota in atopic children. *BMC Microbiology*.

[31] Penders J., Stobberingh E. E., van den Brandt P. A., Thijs C. (2007). The role of the intestinal microbiota in the development of atopic disorders. *Allergy*.

[32] Sokol H., Pigneur B., Watterlot L. (2008). Faecalibacterium prausnitzii is an anti-inflammatory commensal bacterium identified by gut microbiota analysis of Crohn disease patients. *Proceedings of the National Academy of Sciences of the United States of America*.

[33] Rodriguez B., Prioult G., Hacini-Rachinel F. (2012). Infant gut microbiota is protective against cow’s milk allergy in mice despite immature ileal T-cell response. *FEMS Microbiology Ecology*.

[34] Powell R. J., Du Toit G. L., Siddique N. (2007). BSACI guidelines for the management of chronic urticaria and angio‐oedema. *Clinical and Experimental Allergy*.

[35] Szajewska H., Horvath A. (2018). Lactobacillus rhamnosus GG in the primary prevention of eczema in children: a systematic review and meta-analysis. *Nutrients*.

[36] Dhama K., Latheef S. K., Munjal A. K. (2017). Probiotics in curing allergic and inflammatory conditions - research progress and futuristic vision. *Recent Patents on Inflammation & Allergy Drug Discovery*.

[37] Ai C., Ma N., Zhang Q. (2016). Immunomodulatory effects of different lactic acid bacteria on allergic response and its relationship with in vitro properties. *PLoS One*.

[38] Szajewska H., Shamir R., Turck D., van Goudoever J. B., Mihatsch W. A., Fewtrell M. (2015). Recommendations on probiotics in allergy prevention should not be based on pooling data from different strains. *The Journal of Allergy and Clinical Immunology*.

[39] Fleischer D. M., Sicherer S., Greenhawt M. (2015). Consensus communication on early peanut introduction and the prevention of peanut allergy in high-risk infants. *World Allergy Organization Journal*.

[40] Ricci G., Cipriani F., Cuello-Garcia C. A. (2016). A *clinical reading* on “World Allergy Organization-McMaster University Guidelines for Allergic Disease Prevention (GLAD-P): Probiotics”. *World Allergy Organization Journal*.

